# Kaptin-Actin Binding Protein (KPTN)-Related Disorder: A Case Report of Two Siblings Harboring a Novel KPTN Mutation

**DOI:** 10.7759/cureus.98487

**Published:** 2025-12-04

**Authors:** Indrani Biswas, Alex Fleet, Hamza Rastanawie, Shilin Yang, Ali Deif, Peter G Bernad

**Affiliations:** 1 General Practice, KPC Medical College and Hospital, Kolkata, IND; 2 Medical School, University of Cambridge School of Clinical Medicine, Cambridge, GBR; 3 Neurology, Aleppo University, College of Medicine, Aleppo, SYR; 4 Neurology, Huashan Hospital, Fudan University, Shanghai, CHN; 5 Medical School, Khalifa University, College of Medicine and Health Sciences, Abu Dhabi, ARE; 6 Neurology, George Washington University Hospital, Washington, DC, USA

**Keywords:** kptn, kptn-related disorder, myoclonic seizures, neurogenetics, sleep apnea

## Abstract

Kaptin-actin binding protein (KPTN)-related disorder is a rare autosomal recessive neurogenetic syndrome resulting from mutations in the KPTN gene, encoding the actin-binding protein kaptin. Symptoms experienced by individuals with KPTN-related disorder vary, but common features include intellectual disability, developmental delay, psychiatric manifestations, neonatal hypotonia, macrocephaly, and seizures. This case report describes two siblings from the United States affected by KPTN-related disorder, patient B (a 32-year-old woman) and patient M (a 30-year-old man). Both patients B and M have intellectual impairment, have had developmental delay/regression, and exhibit a range of neuropsychiatric symptoms. Additionally, both patients B and M experience seizures, with patient B primarily having absence seizures, and patient M having frequent myoclonic seizures. Interestingly, both patients discussed here maternally inherited a novel KPTN mutation, not previously identified in other individuals with KPTN-related disorder, to our knowledge. Of particular interest in this case is that several members of the maternal family were observed to have myoclonic seizures, raising the intriguing possibility that this newly identified KPTN mutation may lead to mild symptoms even in heterozygous carriers of this mutation. Furthermore, we discuss the recent diagnosis of both patients with sleep apnea, a feature not previously identified in other patients with KPTN-related disorder.

## Introduction

KPTN-related disorder is a rare autosomal recessive disorder resulting from mutations in the KPTN gene on chromosome 19 [1]. A study by Buchert et al. found that KICSTOR subunit 2 (KICS2) loss-of-function variants disrupt KICSTOR complex integrity and mTORC1 nutrient signaling, leading to impaired cilia biology and causing autosomal recessive intellectual disability with epilepsy [2,3]. KPTN forms part of the KICSTOR complex, involved in the regulation of the mTOR signaling pathway [2,3]. The disorder is characterized by moderate-to-severe intellectual disability, global developmental delay, neurobehavioral/psychiatric manifestations (including autism spectrum disorder, anxiety, stereotypies and hyperactivity), neonatal hypotonia, postnatal and progressive macrocephaly, and seizures [4]. Other, less common, symptoms have been reported in patients with KPTN-related disorder, including recurrent infections, conductive hearing impairment, strabismus, nystagmus, ketotic hypoglycemia, thyroid dysfunction, and/or mild skeletal manifestations [1].

KPTN-related disorder is extremely rare, and the precise prevalence of the disorder is unknown. To date, 54 individuals from 32 families with KPTN-related disorder have been identified [1]. Adult phenotypic presentations are particularly underrepresented in the literature, as most published cases involve children. Furthermore, the potential clinical implications of heterozygous KPTN variants remain unclear, and reports of sleep-associated phenotypes are sparse and inconsistent. These gaps present an opportunity to expand the current understanding of this condition, especially regarding its expression in adulthood and the broader phenotypic spectrum. Mutations in KPTN have a higher prevalence within the Amish community. To date, there have been 14 individuals with KPTN-related disorder identified within the Amish community [1].

This case report discusses the cases of two siblings from the United States: patient B (a 32-year-old woman) and patient M (a 30-year-old man), who were both diagnosed with KPTN-related disorder following genetic testing. This report discusses the symptomatic features present in both patients and the identification of a novel genetic mutation within the KPTN gene, not previously reported in other patients with KPTN-related disorder. This report also discusses the identification and management of sleep apnea in these two patients, a feature not previously associated with this disorder. These cases provide rare insight into adult manifestations of this condition and underscore the need for broader recognition of its potential phenotypic variability.

## Case presentation

Patient B

Perinatal and Early Medical History

Patient B is a 32-year-old woman from Virginia, United States of America. In utero, complications began from six months of gestation for which no underlying cause was identified, after which there was a reduction in fetal growth and reduced fetal movement. There were no further complications during pregnancy, with an uncomplicated vaginal delivery at 37 weeks with a birth weight of 6 lbs. At birth, it was noticed that B had a larger-than-usual head. Additionally, immediately after birth, patient B had poor Apgar scores and persistently low oxygen saturation levels, requiring a brief admission to the neonatal intensive care unit (NICU) for supportive care. She was discharged from the hospital after two days. Other complications identified in the neonatal period included hypotonia (her mother described her as a “floppy baby”) and feeding difficulties secondary to a palatal malformation. The palatal malformation was characterized by a high, narrow hard palate with widening of the lower palate as her teeth erupted. This structural configuration impaired sucking and swallowing and led her to chew on the inside of the bottle nipple in an attempt to compensate. Thickened feeds were required to ensure adequate intake and reduce the risk of aspiration.

During early childhood, patient B was enrolled in a special needs physical education program and received ongoing physical therapy due to persistent hypotonia. As she grew older, the hypotonia became more pronounced. At six years of age, she was prescribed bilateral supra-malleolar orthotics - custom ankle-foot braces extending above the malleoli and secured with Velcro straps, to improve stability and lower-limb alignment. She wore these orthotics until approximately nine years of age, during which time she required intensive physical therapy, and the braces were progressively extended higher along the lower legs. At around the age of eight, clinicians at Walter Reed National Military Medical Center determined that the orthotics were having a detrimental impact on her overall function. At nine years of age, the supra-malleolar orthotics were discontinued, and she was transitioned to custom heel-level orthoses recommended by a podiatrist. No further diagnostic evaluation of her hypotonia was conducted during this period.

Developmental Milestones

Developmentally, she sat unsupported by five months, began walking at 16-18 months, and acquired speech at the expected age.

Neurological and Seizure History

At 18 months of age, patient B began having absence seizures, with transient “staring episodes.” At the age of three, she had her first tonic-clonic seizure. Her tonic-clonic seizures resolved at the age of five years, but despite trialing multiple anti-seizure medications, her absence seizures persisted, and continue to the present day, at a typical frequency of 1-2 episodes per week. She currently takes no anti-seizure medication. The cessation of her tonic-clonic seizures coincided with a six-week course of cranial manipulation therapy, performed by a pediatric craniologist aimed at correcting “overlapping skull bones” - a treatment for which there is limited scientific evidence, making it unclear whether the observed improvement was directly related to the intervention or part of the natural course of the condition.

Cognitive and Psychiatric Features

Cognitively, B has an intelligence quotient (IQ) of 69 and an estimated mental age of 14-15 years. She has several neuropsychiatric diagnoses, including autism spectrum disorder (ASD), attention-deficit hyperactivity disorder (ADHD), anxiety, depression, and an expressive-receptive language disorder. She lives with her parents, who receive additional support from caregivers. She lives semi-independently in a basement apartment in her parents’ home, and finds joy and purpose working one half-day per week stocking shelves at a local store.

Medical Comorbidities

Her broader medical history features recurrent infections (such as urinary tract infections (UTIs), otitis media), hypermobility, hyperopia, obstructive sleep apnea (managed with CPAP), and ongoing assessment for tachycardia.

Investigations

Prior to genetic diagnosis, MRIs and EEGs performed on patient B were normal.

Patient M

Perinatal and Early Medical History

Patient B’s younger brother, patient M, is a 30-year-old man. The antenatal period of patient M contained a range of complications, including preterm labor at 24.5 weeks, which was managed with the administration of terbutaline. Amniotic membranes ruptured at 37 weeks, resulting in a rare “dry-birth” at 38 weeks of gestation due to total loss of amniotic fluid. His birth weight was 6 lbs, and he required phototherapy for neonatal jaundice. Aside from these antenatal and perinatal complications, the neonatal period was otherwise uneventful. 

Developmental Milestones

Development progressed normally at first: he sat by six months and walked by 12 months of age. Speech began by 9-12 months, but then regressed from 14 months of age, leading to a complete loss of speech, which only returned around the age of 5-6 years.

Neurological and Seizure History

At 14 months, patient M had his first tonic-clonic seizure. While his tonic-clonic seizures resolved by the age of seven, he began having myoclonic seizures at the age of 11-12 years, which continue to the present day. The myoclonic seizures typically occur 1-3 times per day, and episodes typically last 2-3 seconds. He has no post-ictal symptoms, though he does have symptoms preceding the seizure (described as a sensation coming up the back of his spine), and he is aware when he has had a seizure.

Cognitive and Psychiatric Features

Neurodevelopmentally, M has an IQ of 53, and an estimated mental age of 10 years. Like his sister, M has a range of psychiatric diagnoses, including ASD, ADHD, expressive-receptive language disorder, and post-traumatic stress disorder (PTSD) following a motor vehicle accident. Like his sister, M receives in-home support for daily living tasks, including hygiene, dressing, cooking, and laundry.

Medical Comorbidities

Individuals with KPTN-related disorders may present with joint hypermobility, a factor that can predispose them to musculoskeletal injuries. M had sustained a rupture of the left Achilles tendon. Surgical repair of the Achilles tendon was performed successfully, and the patient is currently recovering. M exhibits pronounced hypermobility, which likely contributed to the injury sustained when he collapsed while running. His past medical history is notable for a bleeding disorder, scoliosis, malformations of the ear canal and paranasal sinuses, degenerative disc disease of the lower lumbar spine, eczema, sleep apnea, and asthma. There are notable systemic features such as mild macrocephaly, heavy eyebrows, and recurrent infections. He is also under investigation for a recently identified fibrinolytic disorder.

Investigations:

Like his sister, M underwent an MRI brain scan and an EEG, both of which were normal. The euglobulin lysis test was shortened, demonstrating fibrin clot lysis in under three hours, which is consistent with increased fibrinolytic activity. Clinicians informed the family that while patient M can form clots, the clots undergo premature dissolution, contributing to both an increased bleeding tendency and susceptibility to clotting abnormalities. Further hematologic evaluation for this fibrinolytic disorder is ongoing.

Genetics and family pedigree: a novel KPTN mutation raising intriguing new possibilities in KPTN-related pathology

**Figure 1:**
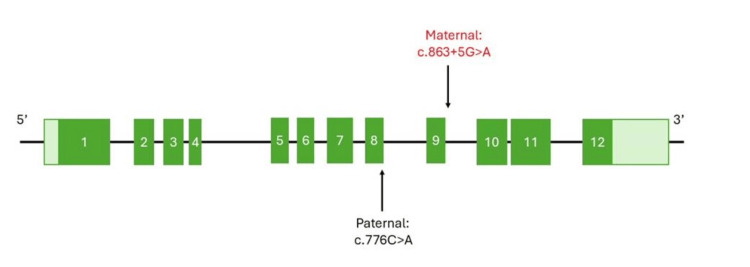
Schematic of the KPTN gene (chromosome 19). The numbered green boxes indicate exons. The location of the mutations in the two individuals discussed in this case report are indicated by arrows. The paternally inherited mutation is located within exon 8, whereas the novel maternally inherited mutation is located within the intron between exons 9 and 10. Figure created using data from National Center for Biotechnology Information (NCBI) Gene.

KPTN-related disorder was diagnosed coincidentally in patients B and M, following an offer of free genetic testing. Their parents have both since undergone genetic testing, helping to provide a more complete picture of the KPTN genetics in this family. Both patients B and M were identified to be compound heterozygotes for KPTN mutations. The mutation inherited from their father is a point mutation in exon 8 of the KPTN gene (c.776C>A), and has previously been identified in other patients with KPTN-related disorder [4]. A case report by Horn et al. [4] identified novel bi-allelic KPTN variants causing autosomal recessive intellectual developmental disorder 41 (MRT41) with developmental delay, macrocephaly, and severe epilepsy, emphasizing variability in seizure severity and suggesting potential benefit from mTORC1 inhibition [4]. The mutation inherited from the mother is a novel mutation within a KPTN intron (c.863+5G>A), which had not previously been identified in other patients with KPTN-related disorder. As a case report, our findings are limited to documenting the presence and inheritance of this intronic variant; functional studies assessing its molecular impact were not available and are beyond the scope of this report. Thus, genetic testing in this case identified a novel genetic mutation associated with KPTN-related disorder. More detail about the location and nature of the KPTN mutations found in patients B and M is shown in Figure 1. 

**Figure 2:**
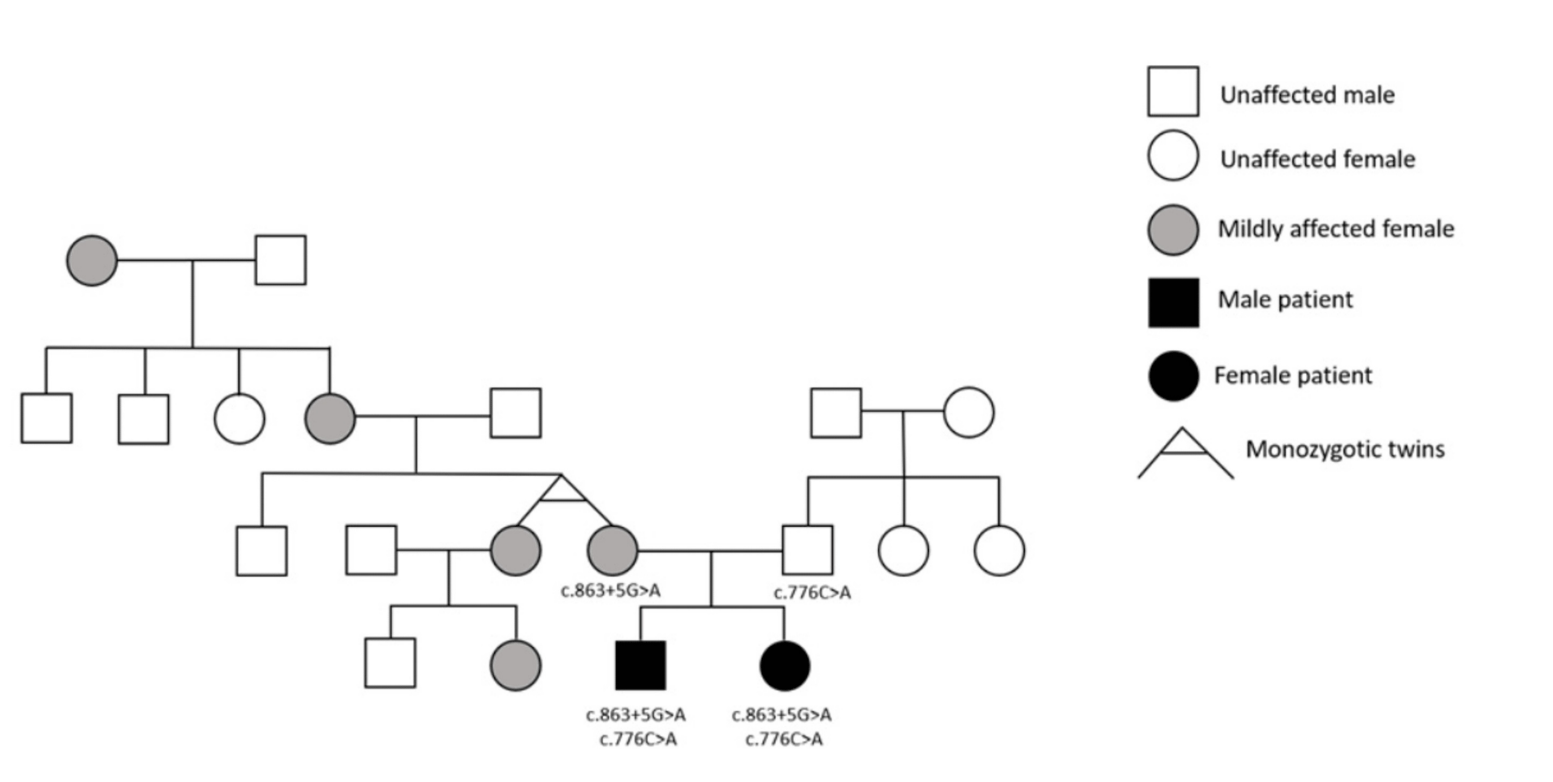
Family pedigree of the two patients described in this case report. The two patients are indicated in black. Individuals who experience myoclonic seizures, but do not have a KPTN-related disorder, are indicated in grey. Individuals who have undergone genetic sequencing are annotated with the identified KPTN mutations.

Of particular interest in this family is the discovery that on the maternal side of the family, from where the novel KPTN mutation has arisen, several family members who do not have KPTN-related disorder do show some mild symptoms, namely myoclonic seizures. However, these observations are purely descriptive and are not intended to imply a confirmed clinical phenotype in heterozygous carriers. This is demonstrated in the family pedigree shown in Figure 2.

As can be seen in the pedigree, several family members on the maternal side have myoclonic seizures only (without other features of KPTN-related disorder), including the patients' mother, her twin sister, the daughter of the mother’s twin sister, and the maternal grandmother and great-grandmother of patients B and M. The patients' mother and her twin sister have a few episodes per month, which have been occurring for as long as they can remember. At present, only the patients' mother has undergone genetic testing to demonstrate that she carries the novel KPTN mutation. The mother’s twin sister is an identical twin, so it would be reasonable to assume that she also carries the novel KPTN mutation. It is not known whether the other individuals who experience myoclonic seizures also carry this novel mutation. Nevertheless, the genetic studies in this family raise the intriguing possibility that even heterozygous carriers of the novel KPTN mutation identified in this family (c.863+5G>A) may show mild symptoms (i.e., episodic myoclonic seizures), despite KPTN-related disorder being an autosomal recessive disease.

These genetic findings demonstrate a compound heterozygous state in both affected siblings and underscore the value of family-based genetic evaluation in rare neurogenetic disorders.

Sleep apnea

Another unique aspect of these cases is the identification of sleep apnea in the two siblings, a finding not reported in other patients with KPTN-related disorder. Patient B was noted by her mother to snore, prompting polysomnography to be performed. The sleep study in April 2024 revealed mild to moderate obstructive sleep apnea (OSA) with an average apnea-hypopnea index (AHI) of 14.8 events/hour, including 77 hypopneas. During rapid eye movement (REM) sleep, AHI increased to 40.9 events/hour. The minimum peripheral oxygen saturation (SpO₂) level was 82%, both overall and during respiratory events, with an average SpO₂ of 91%. No periodic limb movements were observed. A total of one arousal (0.2/hour) occurred during the analysis. The average heart rate was 78 beats per minute (bpm), with a maximum of 158 bpm recorded. Moderate snoring was present, with snorts leading to EEG changes and arousal. Multiple sleep latency testing (MSLT) showed a mean sleep latency of 18.03 minutes, indicating normal daytime sleepiness, and no sleep-onset REM periods (SOREMs) were observed.

In contrast, patient M, despite not snoring, reported significant fatigue and sleep difficulties. Given the family’s seizure history, he also underwent polysomnography, which revealed central sleep apnea. Specifically, the sleep study in April 2024 demonstrated a total of 31 apnea events (apnea index: 3.3/hour) and 13 hypopneas (hypopnea index: 1.4/hour), resulting in a combined AHI of 4.6/hour, consistent with mild sleep apnea. The breakdown included 2 obstructive apneas, 27 central apneas, 2 mixed apneas, and 13 hypopneas, totalling 44 respiratory events. The lowest oxygen saturation (SpO₂) recorded was 92%, with an average SpO₂ of 96%. The average heart rate during sleep was 62.3 bpm, with a peak heart rate of 158 bpm. There were 276 arousals observed (arousal index: 29.1/hour), comprising 19 respiratory arousals, 256 spontaneous arousals, 1 snore-related arousal, and no leg movement or periodic limb movements of sleep (PLMS)-related arousals.

Both were started on full-face CPAP therapy. In both cases, CPAP therapy led to a notable improvement in daytime wakefulness, energy levels, and overall fatigue, reflecting effective management of their respective forms of sleep apnea. However, there was no change in seizure frequency following the intervention, suggesting that while sleep disruption affected energy levels and possibly cognition, it appears not to be a direct driver of seizure activity in these patients. Nevertheless, by reducing their daytime tiredness and improving their daytime energy levels, the identification and treatment of sleep apnea in these patients has led to an overall improvement in their quality of life.

## Discussion

KPTN-related disorder is an extremely rare autosomal recessive neurogenetic syndrome, with approximately 72 affected individuals globally, to our knowledge. The existing literature describes a range of symptoms typically associated with KPTN-related disorder, including intellectual disability, developmental delay, psychiatric manifestations, neonatal hypotonia, macrocephaly, and seizures [5]. A study by Baple et al. showed mutations in KPTN, encoding kaptin, disrupt actin cytoskeleton dynamics, leading to macrocephaly, neurodevelopmental delay, and seizures, establishing kaptin as essential for normal human neuromorphogenesis [5]. The patients discussed in this case report do indeed display several of these characteristic features. Table 1 below summarizes the typical features of KPTN-related disorder and the commonality of these features [1].

**Table 1:** Summary of the most commonly reported symptoms in individuals with confirmed KPTN-related disorder, and the percentage of individuals in which each symptom is present This table is reproduced from Rawlins et al. [1]

Features	% of Persons w/Feature	Comments
Intellectual disability	100%	In general: mild to profound
Developmental delay/regression	93%	
Neurobehavioral/ psychiatric manifestation	84%	Anxiety, stereotypies, impaired social interactions, hyperactivity, repetitive speech
Hypotonia	55%	
Macrocephaly	49%	Postnatal and progressive w/onset in 1st year of life
Seizures	44%	Generalized tonic-clonic, absence, focal/complex partial
Characteristic craniofacial features	84	84% of affected persons have frontal bossing and ≥1 additional feature (short down-slanted palpebral fissures, hypertelorism, depressed nasal bridge, broad nasal tip, tall, broad chin, thick vermilion of lower lip).
Recurrent infections	27%	
Strabismus/nystagmus	12%	
Ketotic hypoglycemia	8%	

In addition to these core features, there are several unique and intriguing features present within the cases discussed here that have not yet been described within the literature, to our knowledge. Beginning with genetics, this report describes a new mutation within the KPTN gene not yet reported within the existing literature. Additionally, analysis of the family pedigree in patients B and M identifies several members of the maternal family who, despite not having a KPTN-related disorder, do have myoclonic seizures. This includes the mother of patients B and M (who carries the novel mutation) and her identical twin sister (who we can assume carries the novel KPTN mutation). This raises the intriguing possibility that, despite KPTN-related disorder being an autosomal recessive disease, heterozygous carriers of the novel mutation identified here may show mild symptoms (specifically, myoclonic seizures). Of course, this cannot be firmly concluded based on the findings in this family alone, as they do not establish a confirmed phenotype in heterozygous carriers. However, if future patients with KPTN-related disorder are identified who also have this novel mutation, it would be fascinating to know whether heterozygous carriers in these families also exhibit some symptoms of KPTN-related disorder, such as seizures. 

Secondly, the cases described here are the first known cases of individuals with KPTN-related disorder who exhibit sleep apnea, to our knowledge. As described above, patient B has been diagnosed with OSA, whilst patient M has been diagnosed with central sleep apnea. This finding was particularly interesting given the well-established association between sleep apnea and seizures. Comorbidity of OSA and epilepsy has been studied for decades, and several authors have reported a high incidence of this comorbidity. In a recent multicenter polysomnography (PSG) study of 166 patients with epilepsy, for example, the prevalence of OSA was 38% [6]. A study by Phabphal et al. [6] found that OSA occurred in 38% of patients with epilepsy, with the NoSAS score showing the highest diagnostic accuracy (Area Under Curve - AUC > 0.7) though not statistically superior to STOP-BANG, STOP-BAG, or SA-SDQ, suggesting that NoSAS may be the most effective screening tool for OSA in epilepsy when used as an ordinal scale [6]. Additionally, a study reported that out of 480 adult patients with sleep apnea syndrome, 4% had seizures [7]. A study by Sonka et al. found that in a cohort of adults with sleep apnea syndrome, 4% had epilepsy, with nearly 80% of seizures occurring during sleep, indicating that sleep apnea and hypopnea may facilitate epileptic seizures [7]. Furthermore, treatment of patients with seizures and OSA with CPAP has been recognised as a means of reducing seizure frequency, although the exact underlying pathophysiology of the comorbidity of OSA and epilepsy and the mechanistic basis for this association are complex and not fully understood [8].

In OSA, hypoxemia and sleep deprivation have been proposed to exacerbate epilepsy [9]. In a study conducted by Goyal et al. [9], rapid eye movement sleep was proposed to be inherently protective against epilepsy due to its characteristic feature, EEG resynchronization, and REM sleep deprivation can potentially lead to epileptogenesis [10]. A study conducted by Jaseja et al. [10] showed that in adults with drug-resistant epilepsy, seizures occurring before or during the primary sleep period were associated with significantly delayed REM sleep onset and reduced REM duration, suggesting a temporal link between REM-sleep disruption and seizure occurrence [10]. Patients with epilepsy tend to have less efficient sleep and shorter REM sleep. Seizures are far more likely to arise from sleep transitions and non-rapid eye movement (NREM) sleep compared to REM sleep [11]. A study by Kilgore-Gomez et al. [11] showed that in adults with drug-resistant epilepsy, REM sleep was significantly delayed and reduced following seizures, supporting a reciprocal interaction between seizure occurrence and reduction in REM duration [11].

As discussed above, the relationship between sleep disorders and epilepsy in the context of KPTN-related disorders has not previously been characterised. Although treating sleep apnea with CPAP did not lead to a change in seizure frequency in the cases of the two siblings discussed here, the importance of assessing patients with seizure disorders for sleep disorders remains an important learning point to be taken from these cases, particularly in rare neurogenetic syndromes where the full phenotypic spectrum is still being defined. As mentioned previously, research involving large groups of patients with both epilepsy and OSA has reported a decrease in seizure frequency following treatment with CPAP, and, as in these cases, patients’ quality of life can still be improved by treating sleep apnea, even if their seizure frequency remains unchanged. 

These cases expand current knowledge of KPTN-related disorder by documenting a novel intronic variant, highlighting the need for further research into potential manifestations in heterozygous carriers, and describing sleep apnea as an observed clinical feature. Although these observations cannot establish causality or broader generalizability, they contribute important preliminary insights that may guide future research into genotype-phenotype relationships, mechanisms of phenotypic variability, and the role of sleep disturbances in this rare disorder.

## Conclusions

This dual case report expands both the scientific and clinical understanding of KPTN-related disorder. Both patients described here maternally inherited a novel KPTN mutation not previously documented in individuals with this disorder, to our knowledge. Interestingly, several maternal relatives who do not have KPTN-related disorder exhibit mild neurological symptoms (i.e., brief myoclonic seizures), raising the possibility of symptom expression in heterozygous carriers. Although KPTN-related disorder is classically defined by autosomal recessive inheritance, these observations suggest potential subclinical effects or variable penetrance associated with specific variants. It highlights an area where further research, including functional studies and broader familial genotyping, may help clarify whether certain variants are associated with subtle or subclinical neurological features. Thus, these findings underscore the importance of genetic testing and expanded familial screening to better define the phenotypic spectrum and functional impact of novel KPTN mutations.

Additionally, these cases have identified obstructive and central sleep apnea as potentially observed features of the condition. Both siblings demonstrated significant improvement in energy and daytime alertness following CPAP therapy, suggesting that treating sleep-disordered breathing may meaningfully impact quality of life, even if it does not directly influence seizure frequency. These insights highlight the importance of proactive sleep evaluations in individuals with KPTN-related disorder (or any seizure disorder), especially when fatigue, disrupted sleep, or daytime somnolence are reported. Together, these cases emphasize the importance of detailed clinical characterisation and continued reporting of rare presentations to advance understanding of this uncommon disorder.
